# Precise prediction of the radiation pneumonitis in lung cancer: an explorative preliminary mathematical model using genotype information

**DOI:** 10.7150/jca.37708

**Published:** 2020-02-10

**Authors:** Lehui Du, Na Ma, Xiangkun Dai, Wei Yu, Xiang Huang, Shouping Xu, Fang Liu, Qiduo He, Yanli Liu, Qian Wang, Xiangtao Liu, Hui Zheng, Baolin Qu

**Affiliations:** 1 Department of Radiation Oncology, Chinese PLA General Hospital, Beijing, 100853, P.R. China.; 2 Tianjia Genomes Tech CO., LTD., Hefei, 238014, P. R. China.

## Abstract

**Purpose**: Radiation pneumonitis (RP) is the most significant dose-limiting toxicity and is one major obstacle for lung cancer radiotherapy. Grade ≥2 RP usually needs clinical interventions and serve RP could be life threatening. Clinically, tissue response could be strikingly different even two similar patients after identical radiotherapy. Previous methods for the RP prediction can hardly distinguish substantial variations among individuals. Reliable predictive factors or methods emphasizing the individual differences are strongly desired by clinical radiation oncologists. The purpose of this study is to develop an approach for the personalized RP risk prediction.

**Experimental Design**: One hundred eighteen lung cancer patients who received radiotherapy were enrolled. Seven hundred thousand single-nucleotide polymorphism (SNP) sites were assessed via Generalized Linear Models via Lasso and Elastic-Net Regularization (GLMNET) to determine their synergistic effects on the RP risk prediction. Non-genetic factors including patient's phenotypes and clinical interventional parameters were separately assessed by statistic test. Based on the results of the aforementioned analysis, a multiple linear regression model named Radiation Pneumonitis Index (RPI) was built, for the assessment of Grade ≥2RP risk.

**Results**: Only previous surgery and fractional dose were discovered statistical significantly associated with grade ≥2RP. Thirty-nine effective SNPs for predicting the Grade ≥2RP risk were discovered and their coefficients of the synergistic effect were determined. The RPI score can successfully distinguish the RP≥2 population with 92.0% sensitivity and 100% specificity.

**Conclusions**: Individual radiation sensitivity can be determined with genotype information and personalized radiotherapy could be achieved based on mathematical model result.

## Introduction

Lung cancer is one of the most common cancers in the world that has a poor prognosis with the 5-year survival rate of less than 18%.^1^ According to the estimation from the World Health Organization (WHO), lung cancer will cause about 2.09 million annual cases of death worldwide, making it a leading cause of cancerous death (https://www.who.int/news-room/fact-sheets/detail/cancer). Radiotherapy (RT) is one of the major treatments for lung cancer. The main restriction for lung cancer radiotherapy is radiation-induced lung injury (RILI). Although radiologists already took advantages of modern radiation techniques to minimize the injury of normal tissue, pulmonary toxicity has always been an obstacle for them that cannot be bypassed.^2^ The same restriction applies to the treatments of other thoracic malignancies, like esophageal cancer.

RILI includes acute radiation pneumonitis (RP) and chronic lung fibrosis. Depending on the methods of assessment, it has been estimated that about 5% to nearly 40% of lung cancer patients who underwent radiotherapy will develop RILI.^2^ Since lung is a very radiosensitive organ, radiation pneumonitis can occur in a short period and lead to pulmonary insufficiency. Grade≥2 RP usually needs clinical interventions and about 10%-20% of them are severe RP (grade≥3). Once a severe acute radiation pneumonitis occurred, the symptom usually will progress very rapidly, could be irreversible, and might turn into a life-threatening syndrome. Unfortunately, once the severe RP was developed limited clinical interventions could be applied to control the condition. Therefore, it is of importance to estimate the RP risk, particularly for the RP that needs interventions.^3^ An early prediction will help clinicians actively take interventional measures an reduce the risk of patients. Although 13-100% patients after the RT could be found had radiological signs of RILI in the images by computed tomography (CT), single-photon emission computerized tomography (SPECT), or magnetic resonance imaging (MRI), much less fraction of these patients will actually develop clinical symptoms.^2^ Thus, reliable predictive methods are strongly desired by clinical radiologists.

Tissue response to the radiation is a complex pathophysiological process and multiple factors might influence the symptoms. These factors include treatment factors like dosimetric parameters, physiologic factors such as age or gender, and genetic factors like genetic variants that confer radiosensitivity. From the previous studies, the suggested multiple predictive factors for the RP risk include dosimetric parameters, age, gender, smoking history, and cytokines like TGF-bete1 etc. Models that combined multiple clinical and dosimetric variables were also suggested, such as Lyman model and its improved version, Transfer Factor Spared from receiving >5 Gy model, and QUANTEC model.^4^-^12^ However, there was still inconsistent opinions about the power of each interventional parameters and no consensus was achieved about the best appropriate approach for RP risk assessment. More importantly, one problem that is difficult to solve for radiation oncologist is that two similar patients received identical radiotherapy could response significant differently. The underlying reason can be the difference of radiosensitivity between the two patients, which is determined by genetic. Unfortunately, most of the previous studies, the effects of genetic variations have been largely ignored. Only one model incorporated the patient's genetic information into RP prediction but focused on limited genetic variants.^11^

To address the issue of radiation-induced pulmonary toxicity, particularly on the individual variances of pneumonitis, we systematically assessed a large number of genetic variants as well as other factors that could potentially affect the risk of developing RP. Our results showed that the dosimetric parameters and other clinical factors played a much less important role than expected while genetic variations were much more suggestive. Based on these results, we built a mathematical model that can predict the risk of developing an RP that needs clinical interventions (grade ≥2). The algorithm just needs personal genotype information and it can help radiologists accurately assess the risk even before the radiotherapy.

## Materials and Methods

### Patients

In this prospective study, a cohort of 118 newly diagnosed lung cancer patients with definitive radiotherapy was recruited from April 2016 to March 2018 at the Chinese PLA General Hospital (Beijing, China). The eligible criteria were as following: histologically or cytologically confirmed lung cancer including non-small cell lung cancer and small cell lung cancer; no severe radiotherapy contraindications (including severe cardiopulmonary disease, severe autoimmune diseases, pregnant or lactating women, etc.); no previous and coexistent thoracic radiotherapy; Pulmonary function tests for patients before radiotherapy were strongly recommended but not compulsory; Karnofsky performance status (KPS) ≥60 scores. Symptom evaluation for each patient was required in advance; Patients' characteristics and their outcomes were unknown to investigators performing genetic analysis. Genetic analyses were independent of clinical practice. The research was approved by the Internal Review Board of Chinese PLA General Hospital (Beijing, China) and consent was obtained from all patients enrolled.

### Radiation treatment

All patients were treated with image-guided intensity-modulated radiation therapy (IMRT) with 6-MV X-rays from the two predefined linear accelerators (Elekta Synergy and Varian clinic ix). Radical and palliative treatment with a total dose of 30-72Gy was performed once a day, five days per week. A computed tomographical simulation was performed before radiotherapy treatment. Target volumes and critical normal organs were delineated by the three-dimensional Pinnacle planning system (version 9·2, Philips). Basic clinical characteristics and treatment details of those patients are shown in Table 1.

### Pulmonary toxicity assessments and follow-ups

All patients recruited were checked and were evaluated prospectively by their radiation oncologists weekly during radiotherapy and 4-6 weeks after treatment completion. Follow-ups were performed every 6 weeks for the first 3 months and thereafter every 3 months. Extra visits were required if symptoms showed up. Radiographic examination by chest X-ray or computerized tomography was performed at every follow-up visit. RP was diagnosed by clinical manifestations (e.g. dyspnoea, cough, pain and low-grade fever) and radiological findings. Once diagnosed, RP was further graded by at least two radiation oncologists following the Common Toxicity Criteria for Adverse Events (CTCAE) version 4.03. If the symptoms were present at baseline, worsening of symptoms of at least one grade was considered as RP. In addition, if there was pulmonary infection or thoracic disease progression, RP was excluded from the diagnosis. The diagnosis of RP of grade ≥ 2 was defined as the primary end point. If symptoms were present at baseline, worsening at least one grade was considered as RP. The following situations were excluded when diagnosis the RP: (1) pulmonary infection; (2) thoracic disease progression (PD). Criteria for each grade were as follows. Grade 0, no change. Grade 1, RP was asymptomatic and can only be observed in radiographic findings. No intervention was indicated. Grade 2, patient manifested symptoms that limit activities of daily living (ADL). Medical intervention has been indicated. Grade 3, patient manifested severe symptoms limiting self-care ADL. Oxygen has been indicated. Grade 4, patient manifested life-threatening respiratory compromise with urgent intervention indicated (e.g. tracheotomy and intubation). Grade 5, RP has caused lethality. Treatment periods started with the initiation of radiotherapy, and the patients were censored until last follow-up or death.

### DNA extraction and genotyping

Peripheral blood leukocytes from patients before the radiotherapy was used for genomic DNA extraction using the Maxwell system (Promega, Madison, WI, USA). Genotypes of ~70,000 sites were determined by Infinium® Global Screening Array system (Illumina, San Diego, CA, USA) following the manufacturer's instruction.

### Statistical analysis

The association between patient characteristics and variables were separately assessed by MannWhitney U test for continuous variables (i.e. age) and Fisher-exact test categorical variables (i.e. Gender).

### Quality control

We excluded SNPs in each individual dataset that had a mean GenCall score < 0.7, missingness >5%, MAF < 0.01 or a Hardy-Weinberg equilibrium test P < 10^-6^ using PLINK. We also excluded variants with multiple alleles. A total of 720,078 SNPs in the genotypic data set and 299,054 SNPs in the dataset passed this process for further prediction.

### Initial value assignment

Genotyping result of each site was converted into a numerical value as the initial assignment of that site. Basically, the genotype on the human standard reference genome (version 37, GRCh37) was used as reference genotype and was referred as 'wild type'. Patient's genotype was compared with the reference genome. If the sequence of the allele was the same as the wild type, the initial assignment for that allele will be '0'. If the sequence was different from the wild type and was the alternative sequence on the standard reference genome, the initial assignment for that allele will be '1'. If the allelic sequence was different from the wild type and was not the alternative sequence on the standard reference genome, the initial assignment for that site will be '0'. The sum of two allelic values will be the initial assigned value for that site. This value is an integer varies from 0 to 2 (See supplemental method for detailed method and scripts for computer programing).

### Model developing strategy

Our goal is to build a mathematical model that can predict the risk of RP that needs interventions. Although we believe that the susceptibility of a tissue to the radiation is mainly determined by the genotypes, we cannot presumably exclude the impact of non-genetic factors (phenotypes and dosimetric parameters), since developing RP is a very complex process. First, we assessed the relevance of the non-genetic factors to the RP risk using statistical tests. If any of these factors showed statistical relevance to the RP risk, we would incorporate this factor into our model. Because there are too many genetic variants (genotypes), we decided to directly apply regression analysis to the data set and to check if the desired result can be acquired. Briefly, the data was split into two parts, one part for the training and the other part for the validation. To obtain a more reliable model, the majority part of the data was used for the training set. Ninety sets of data were randomly selected from the pool for the training and the rest 28 sets of data, which were also random data, were used for the validation.

### Coefficients for the model

The diagnostic model of RP was made with a multivariate linear model approach based on the Elastic Net algorithm implemented in the R (version 3.5.1) package ''glmnet''. This approach is a combination of traditional Lasso and ridge regression methods, emphasizing model sparsity while appropriately balancing the contributions of correlated variables. It is ideal for building linear models in situations where the number of variables (markers) greatly outweighs the number of samples. Optimal regularization parameters were estimated via 10-fold cross-validation. Bootstrap analysis was employed sampling the data set with replacement 500 times and a model for each bootstrap cohort were built. Only markers that were present in more than half of all bootstraps were included in the final model. The covariates age, dose, fractional dose, V5, V10, V20, V30, and MLD were included in the model and were exempted from penalization (regularization).

## Results

### Patient characters

Archived information of 118 lung cancer patients was obtained from the People's Liberation Army General Hospital. All patients received IMRT therapy from June 2015 to May 2018. The case information of 118 lung cancer patients, including 102 men (86·5%) and 16 women (13·5%) was listed in Table 1. The median age of patients was 60 years old (age ranges from 36 to 79 years old). In terms of histology, 41 (34·7%) out of the 118 patients were diagnosed with squamous cell carcinoma, 19 (16·1%) with adenocarcinoma, and 53 (44·9%) with small cell lung cancer. 86 cases (72·8%) had stage III lung cancer, 25 cases (21·2%) in stage IV, and 7 patients in stage I-II. Of all patients, 82·2% were current or former smokers. Forty patients (33·9%) were also diagnosed with the chronic obstructive pulmonary disease (COPD). Notably, 10 patients (8·5%) had undergone surgery before radiotherapy. Most patients (96·6%) were treated with a combination of radiotherapy, chemotherapy, or targeted therapy. The median radiation dose delivered to the primary tumor was 61·6Gy (range 30-70Gy). The median values for MLD, V30, V20, V10 and V5 were 11·6 Gy (range 3·3-19Gy), 12% (2·0%-23·1%), 20% (2·0%-30·5%), 34·2% (4·0%-60·0%) and 51·0% (13·8%-86·0%), respectively. The median follow-up time was 314 days (range 37~614 days) after the beginning of radiotherapy. Among the 118 patients, 50 patients (42·4%) developed RP of grade ≥2. The median interval to RP (grade ≥ 2) diagnosis was 86 days (range 33-205 days).

### Clinical and other non-genetic factors

Non-genetic factors including age, gender, COPD status, smoking status and multi dosimetric parameters from all 118 patients were firstly assessed for their relevance to the RP. Based on clinical relevance, instead of classifying whether the patient will develop the RP, we classified patients as whether they will develop an RP that needs clinical intervention, i.e. whether RP grade ≥2. The p-values of Mann-Whitney U test for continuous variables and Fisher-exact test for categorical variables were calculated for each individual factor. The statistical comparison of the clinic parameters of major clinic parameters were shown in Table 2. Among all clinical parameters, only one parameter, previous history of surgery showed statistical significance, which was in consistent with previous report.^13^ As for other non-genetic parameters particularly the most important dosimetric parameters, the only factors that showed statistical significance is the fractional dose (Table 3), with the p-value of 0.029.

### Genetic variations

**Figure 1:**
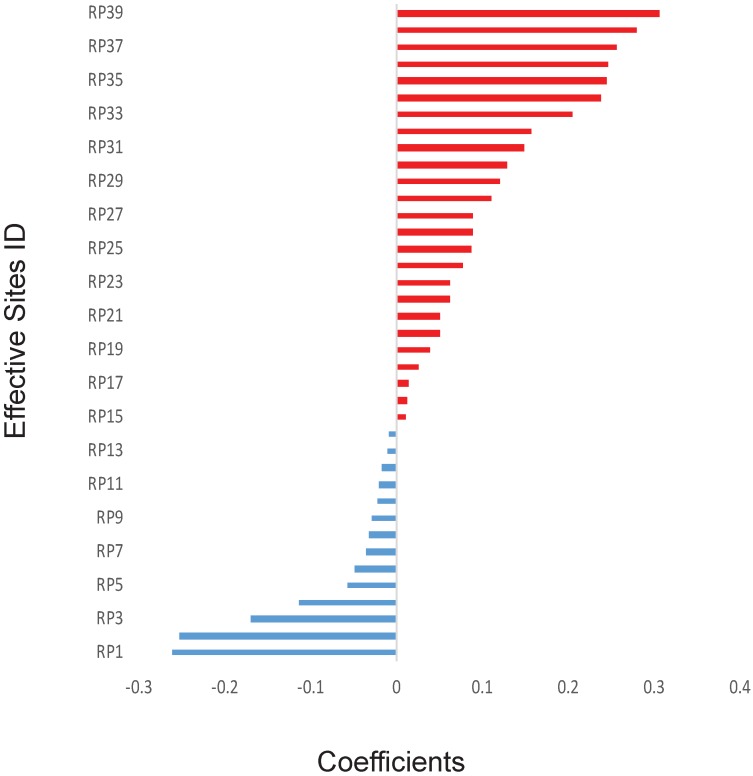
Calculated effects of SNPs. The coefficients of 39 SNP sites were shown for their synergistic powers on the prediction of RP ≥2. The SNP sites were shown only when absolute effects are greater than zero using Elastic net and generalized linear model. X-axis: Correlation coefficients. Y-axis: IDs of the SNP sites.

The previous works have shown some specific genes, such as TP53, may play a key role in the outcomes of radiotherapy. However, in this study, to avoid introducing any artificial bias in our model, non-biased initial values were assigned for all the 700,000 sites, including important sites reported in the literature.^11^,^14^ Because there are too many of them (over 299,000 sites after quality control) and each of them might have only minor effect with unknown dependence on other genetic factors. Therefore, it will be inappropriate to do statistical analysis on those genetic factors one by one. Thus, we directly applied the mathematical model on those factors. Thirty-nine effective SNP sites were discovered after applying the GLMNET regression on 90 sets of random training data. The exact locations of these 39 sites were shown in Supplementary Table 2 and the coefficients of these effective sites were illustrated in Figure 1. Precise coefficients of these 39 sites were also included in the Supplementary Table 2. Among the 39 sites, 14 of them had a negative coefficient and 25 were positive with value varying from about -0·26 to about 0·31 (Figure 1).

### Radiation pneumonitis index

Since our result showed that non-genetic factors may not play key roles for the RP development, only genotype data were used to build the predictive model for the grade≥2 RP risk. We compared genotype information of 39 sites discovered by GLMNET with the stand human reference genome (GRCh37). If the allele is the same as reference, we defined it as wild-type and abbreviated as 'W'. If the allele is the same as the alternative in the stand reference genome, we defined it as alteration and abbreviated as 'A'. Therefore, the genotyping result for each site can be converted to 'WW', 'WA'/'AW' or 'AA'. We assigned value 0 to 'WW' genotype, value 1 to 'WA'/'AW' genotype and value 2 to 'AA' genotype. By combining the assigned value and coefficient values of each site, a Radiation Pneumonitis Index (RPI) was defined as:

RPI=Pr (A_i_*C_i_)

Where: A_i_=assigned value of the site i, which equals “0” when genotype is homozygous of “wildtype”, or equals “1” when genotype is heterozygous of “wildtype” and “alterate”, or equals “2” when genotype is homozygous of “alterate”; C_i_ = coefficient value of the site i, the detailed value for each site see **Supplementary Table 1.**

i.e. RPI=Pr(-0.2603*(RP1 value)-0.25247*( RP2 value)+… +0.279084*(RP37 value)+ 0.305607*(RP38 value)),

Pr is the probability obtained from the GLMNET algorithm.

**Figure 2:**
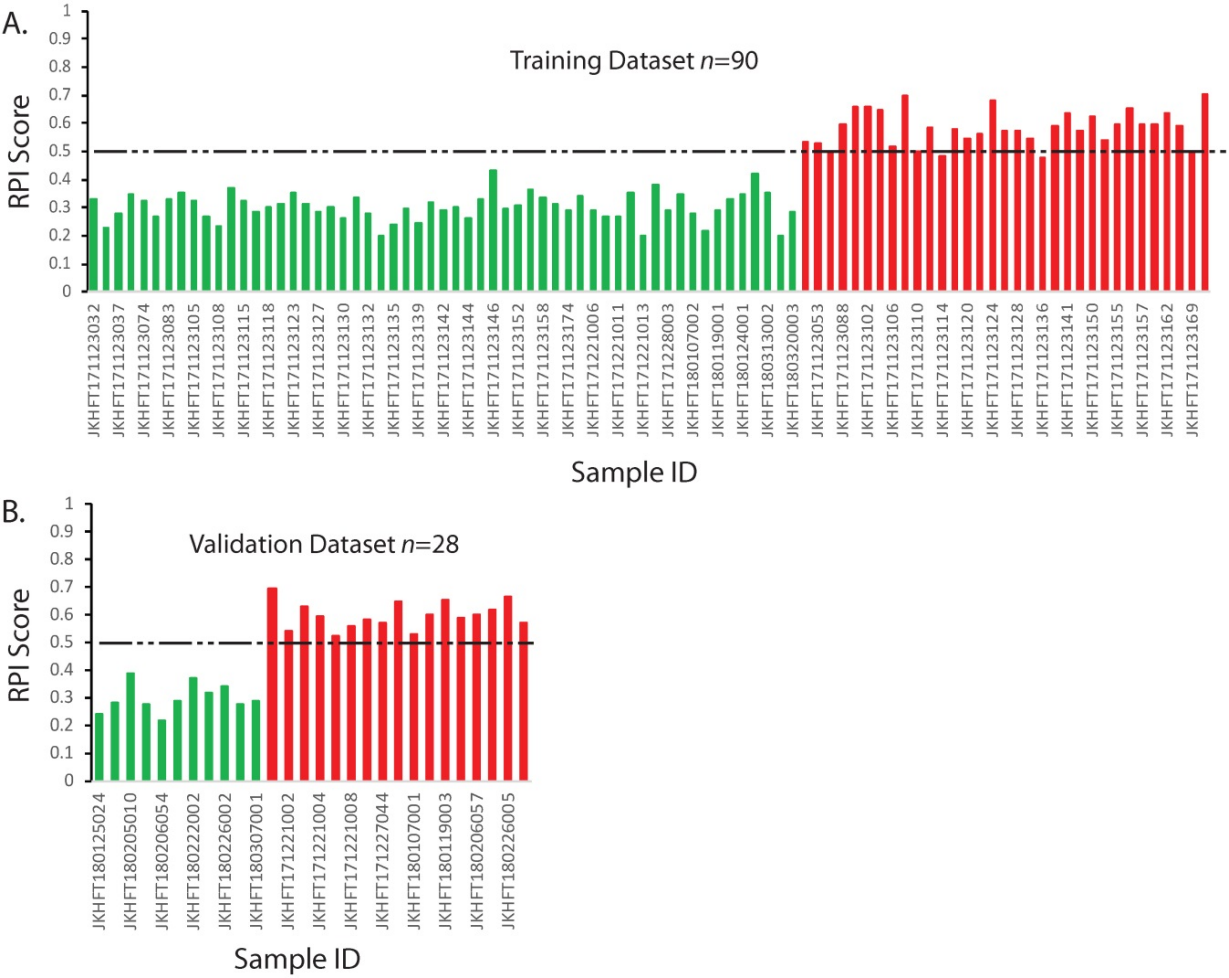
Prediction model and validation for RP ≥2. The RPI scores of each sample were calculated according to the multivariate regression model. The categories of two types of patients were color indexed (RP<2, green; RP≥2 red). Dashed line indicates a threshold that separates two groups. A. Training dataset. B. Validating dataset. X-axis: Sample IDs. Y-axis: RPI Scores

The RPI is a numerical value that can predict the risk of developing a grade≥2 RP based on multiple linear regression algorithm using genotype information. In our training data set, if using the threshold value 0·5 for the RPI value, all the patients who had no RP or developed a grade1 RP could be identified. Among those who had grade≥2 RP, only 4 out of 33 who had marginal scores were falsely classified (Figure 2A). This result demonstrated in the 90 sets of training data, the RPI value could be used to differentiate the patients that have the RP grade ≥2 with 87·9% sensitivity and 100% specificity.

### Validation

Based on the aforementioned model, the RPI values were calculated for the 28 random validating samples. The results were illustrated in the Figure 2B. As shown in Figure 2, all 17 grade ≥2 RP patients had significantly higher RPI value than those who had no RP or grade1 RP. If also using the threshold value 0·5 for the RPI value, the RPI score can distinguish the grade≥2 RP and the RP<2 populations with 100% sensitivity and 100% specificity in our validation dataset. If combined two sets of data together, the overall sensitivity and specificity of RPI model would be 92·0% and 100%, respectively.

## Discussion

Radiation-induced pneumonitis is a major obstacle for the radiotherapy for the thoracic malignancies, particularly lung cancer. One common question asked by the clinical radiation oncologists is why two similar patients who underwent almost exact same radiotherapy developed completely different pneumonitis symptom. We believe the answer exists in the genetic variations and the problem could be solved by the genetic test. Here we introduced a method that can accurately predict the risk of developing the RP that needs interventions even before the therapy. In our study, the statistical results indicated phenotypic factors like age, gender, or smoking history and interventional factors such as dosimetric parameters might not be dominant factors for predicting individual variances of RP risk, which seemed to be against intuition and was inconsistent with the results in the previous literature^4^,^5^,^15^-^18^, particularly when the dosimetric parameters considered. Indeed, the only significant clinical parameter is the chest surgery history before the radiotherapy and the reason is understandable since the tissue with preexisted lesion will be more vulnerable. The reason for such kind of result could be that the maturation of therapeutic regimen and the technological advancement made the variations of these parameters among individuals minimized to a non-significant level. Nowadays most radiotherapeutic plans gave each patient a very similar overall dose and total fractions, and the sophistical delivery technique made other parameters like MLD, V20 or V30 also less diverse. Therefore, the variances of dosimetric parameters became minimized among patients and the decrement of these variances made the dosimetric factors less relevant to the personalized response to the radiation. Our results are by no means unprecedented in the field. Indeed, other researchers also found similar results in their studies.^19^ One interesting discovery on the dosimetric parameters is that the fractional dose shows some significance (p value= 0.02936, Table 3) although the real difference between the G≥2 RP and G<2 RP is very subtle. It is hard to fully assess the meaning of this finding at the current stage because the fractional dose used in this study is calculated dose. There always is a variance between the calculated dose and the real delivered dose. Since the variance of fractional dose between two groups is so small that the difference may be smaller than the variance between the calculated dose and the real delivered dose, we could not exclude the possibility that in the reality the significance of fractional dose is not as strong as calculated in this study because of error. However, we will keep monitoring this point in future studies and will collect more precise data for validation. The unified therapeutic regimen assumes every patient will have a similar radio-sensibility, nevertheless, clinically there does exist significant responsive variances such as the development of RP, and these variations of individual response have turned into a clinical problem.

Clinicians already realized that the genetic variations may play important roles in this issue and have started working towards this direction. Previously, several groups including ours studied the association between the single nucleotide polymorphism (SNP) site and the RP risk.^20^-^24^ However, these studies focused only on few genetic variants and lacked a comprehensive assessment of the synergistic effect of multiple variants. The isolated results from these studies could barely be used for clinical guidance while the complexity of human genome brought many difficulties for solving the problem methodologically. To overcome this drawback, a systematic study on large number of SNPs was performed in this study. For each patient, about 700,000 sites, which covered the whole area of the human genome, were scanned to discover possible RP relevant genetic variations. The goals of the study were to: 1.) discover as many as possible variants that are relevant to the RP risk; 2.) quantitatively determine the effects of these effective variants; 3.) build an accurate model for the RP risk prediction that can be easily used by the radiation oncologists in the clinic. Multiple linear regression model is a model that can fit in many scenarios and was also used in this study for the RP risk prediction.

There are several methods to build the multiple linear regression model, as each method is suitable for a given data set with specific features. The least squares regression is the most commonly used method for multivariate linear regression. If there is a significant linear relationship between the response variable and the predictive variable, the least square regression will have a very small bias. Especially, if the observed number of samples *n* is far greater than the number of predictive variables *p*, the least square regression will have a smaller variance. However, if *n* is close to *p*, it is prone to overfitting; if *n*<*p*, least squares regression cannot get meaningful results. In addition, many variables in the multivariate linear regression model may be independent of the response variables, and there may be multiple collinear phenomena. These situations will increase the complexity of the model and weaken the explanatory power of the model. This requires variable selection (feature selection). The character of our data is a small sample population (118 samples) and a high number of independent variables (about 700,000).it is obvious that *n*<<*p*. In view of the above problems, Robert Tibshirani and others introduced the shrinkage method.^25^ It is mainly ridge regression and lasso regression. By adding penalty constraints to the least squares estimate, some coefficients are estimated to be zero. Elastic net combines two regularization methods, ridge regression, and lasso regression. Elastic net has an obvious better effect on *p* than *n* or severe multi-collinearity. We used Generalized Linear Models via Lasso and Elastic-Net Regularization (GLMNET) to build our algorithm, which is known to work better for the data that has much more independent variables than dependent variables, or data has serious multiple collinearities.^26^-^30^ This algorithm just fitted the data type of our kind, and the final model was validated well.

The basic idea for precision medicine is that each therapy should be tailored to the personal characteristics, which may include congenital features and acquired characters. Genetic features are the most widely used personal characteristics for precision medicine. Medical oncologists have taken advantage of both congenital genetic features (e.g. germline mutations of breast and ovarian cancers) and acquired genetic characters (e.g. somatic mutations of lung cancer etc.) to make cancer therapies advanced into targeted therapy and immunotherapy.^31^,^32^ However, in the field of radiotherapy, much less attention has been paid on the genetic features and much work need be done to make personal genetic features as a guiding biomarker for the clinical practice of radiologists. Here we attempt to develop a kind of precision medicine approach that can help radiation oncologists take advantage of genetic features. Comparing with other methods, our approach is relatively simpler and clinically more practicable. The method just needs the blood sample, which is easy to access, and the genotype information, which can also be easily acquired either by fluorescent PCR, or by gene chip genotyping or by sequencing. The algorithm for the RPI is also straightforward and can be integrated into a simple program. The whole method is time-saving and cost-efficient.

In an early study, Barnett et al performed a research work trying to validate the associations between the previously reported genetic variations and the radiation toxicity in a large independent dataset.^33^ However, the overall results of their study were negative and none of the previously reported association were confirmed. There could be multiple causes for the failure of the confirmation. One possible reason we believe is that the radiotoxicity is a complex pathophysiological process and it is unlikely to predict such a complex process with just single or few genetic variants within limited signal pathways. That is also the problem what we are trying to solve in this study. We believe the ultimate solution should be a model combining multiple variables with the synergistic effect of all factors considered.

We hope other researchers for the precision medicine studies can gain inspirations from the experience we learned in this study and can explore deeper in this field. The type of data we used in this study is very commonly encountered in clinical researches and we believe this method is suggestive and other clinicians can draw on the experience of. Though the sample population in our study is limited and more samples are still needed to stabilize our model, we believe our work explored a new direction for other clinicians and wish this work would also help the advancement of radiogenomics.

## Conclusions

The dosimetric parameters may not play dominant roles in the individual difference of radiation pneumonitis risk for the patients with definitive radiotherapy while the genetic variations are more relevant. A model or an algorithm that incorporates multiple genetic variations is more effective than a single biomarker for the risk assessment and should be the direction in future.

## Supplementary Material

Supplementary table and data.

## Figures and Tables

**Table 1:** Patient Information and Dosimetric Parameters

	Histology/ Stage	Patients (n)	Median	Percentage/ Range
**Male**		102		86·5%
**Female**		16		13·5%
**Age**			60 years	36-79 years
**Histology**	squamous	41		34·7%
	adeno	19		16·1%
	small cell	53		44·9%
	other	5		4·2%
**Stage**	I-II	7		5·9%
	III	86		72·9%
	IV	25		21·2%
**Smoker**		97		82·2%
**Non-smoker**		21		17·8%
**COPD**		40		33·9%
**With former surgery**		10		8·5%
**With chemotherapy or targeted therapy**		114		96·6%
**Primary tumor dose**			61·6 Gy	30-70 Gy
**MLD**			11·6 Gy	3.3-19 Gy
**V30**			12·0%	2·0%-23·1%
**V20**			20·0%	2·0%-30·5%
**V10**			34·2%	4·0%-60·0%
**V5**			51·0%	13·8%-86·0%
**Follow-up time**			314 days	37-614 days
**RP grade≥2 patient**		50		42·4%
**median interval to RP grade≥2 diagnosis**			86 days	33-205 days

COPD= chronic obstructive pulmonary disease; MLD= mean lung dose in Gy; V30, V20, V10, V5= the percentage of the lung volume (with subtraction of the volume involved by lung cancer) which receives radiation doses of 30, 20, 10, 5 Gy or more.

**Table 2:** statistical comparison of the clinic parameters between patients with and without G≥2 radiation pneumonitis

	Patients with RP≥2 (need clinical interventions)		Patients with RP<2 or no RP (do not need clinical interventions)		*p*-value
**Patients (n)**	50		68		N/A
**Male (percentage)**	41(82.0%)		61 (89.7%)		0.2803
**Female (percentage)**	9 (18.0%)		7 (10.3%)		
**Age (median)**	46-79 (61)		36-77 (58)		0.08805
**Histology**	Non-small cell	25 (50.0%)	Non-small cell	35 (51.5%)	0.3102
	small cell	20 (40.0%)	small cell	31 (45.6%)	
	other	5 (10%)	other	2 (2.9%)	
**Stage**	I-II	3 (6.0%)	I-II	3 (4.4%)	0.946
	III	36 (72%)	III	50 (73.5%)	
	IV	11 (22%)	IV	15 (22.1%)	
**Smoker**	10 (20.0%)		11 (16.2%)		0.6319
**Non-smoker**	40 (80.0%)		57 (83.8%)		
**With COPD**	20 (40.0%)		20 (29.4%)		0.2444
**KPS**	80-90(88.6)		70-90(88.38)		0.7955
**With former chest surgery**	9 (18.0%)		1 (1.5%)		0.001854
**With chemotherapy or targeted therapy**	49 (98.0%)		64 (94·1%)		0.5647

COPD= chronic obstructive pulmonary disease; KPS= Karnofsky Performance Scale.

**Table 3:** statistical comparison of the dosemetric parameters between patients with and without G≥2 radiation pneumonitis

	Patients with RP≥2 (need clinical interventions)	Patients with RP<2 or no RP (do not need clinical interventions)	*p*-value
Patients (n)	50	68	N/A
Primary tumor dose (median)	28.6-70 (60.8)	22-70 (61.6)	0.3403
MLD (median)	14.6-1800.5 (1117.0)	12.1-1900.4 (1200.1)	0.2154
V30 (median)	2.02-22.27 (11.93)	2.00-23.05 (12.89)	0.4908
V20 (median)	2.00-30.00 (19.99)	6.02-30.54 (19.90)	0.7315
V10 (median)	4-59.96 (34.45)	14-55(33.47)	0.8831
V5 (median)	12.75-86 (50.07)	3-86(53.43)	0.8424
Fractional dose (median)	2.00-4.67 (2.40)	2.00-7.00 (2.45)	0.02936

MLD= mean lung dose in cGy; V30, V20, V10, V5= the percentage of the lung volume (with subtraction of the volume involved by lung cancer) which receives radiation doses of 30, 20, 10, 5 Gy or more.
