# Supplementary material for: Precise prediction of the radiation pneumonitis in lung cancer: an explorative preliminary mathematical model using genotype information
Source: J Cancer. 2020 Feb 10;11(8):2329–38. doi: 10.7150/jca.37708 (PMC7052914; doi:10.7150/jca.37708)
Supplement: Supplementary file 1 — Supplementary table and data. [file jcav11p2329s1.pdf]

Supplemental Table 1: effective SNP sites

ID	Chr	Location	Coefficients
RP01	18	9102509	-0.2603
RP02	9	138440562	-0.25247
RP03	4	42344051	-0.16969
RP04	6	23434028	-0.11298
RP05	10	54519150	-0.05693
RP06	3	103854896	-0.04958
RP07	2	137866132	-0.03632
RP08	2	222340239	-0.03231
RP09	19	8789227	-0.02924
RP10	8	6517040	-0.02235
RP11	14	48430295	-0.02076
RP12	22	45970957	-0.01783
RP13	9	18861021	-0.01148
RP14	4	117630240	-0.00927
RP15	6	29434414	0.010866
RP16	9	5420526	0.013204
RP17	3	187908176	0.014126
RP18	2	69880314	0.025025
RP19	9	138373864	0.039429
RP20	16	81461322	0.050278
RP21	11	81715144	0.050524
RP22	6	144586746	0.061824
RP23	2	137321430	0.062806
RP24	4	5629298	0.077442
RP25	1	95330745	0.086455
RP26	1	236340179	0.088164
RP27	3	165790002	0.088631
RP28	6	106559602	0.110807
RP29	9	33104540	0.120775
RP30	4	164617773	0.128903
RP31	16	83461553	0.148922
RP32	11	101605466	0.156021
RP33	14	55076477	0.204155
RP34	7	84365672	0.237402
RP35	18	43943801	0.24467
RP36	18	6503627	0.246517
RP37	17	70810416	0.256039
RP38	20	60385967	0.279084
RP39	9	5389652	0.305607

ID is the assigned name for the single nucleotide polymorphism (SNP) that was used for the RP prediction.

Chr is the chromosome on which the corresponding **RPxx** located.

Location is the exact physical coordinate where the corresponding **RPxx** located based on reference genome is GRCh37.p13.

Coefficient is the constant for the multiplying in the algebraic expression of RPI calculation.

Supplemental material, R script for genotype conversion

```
dir="user define"
setwd(dir)
 pheno=read.table("pheno_rp2.txt",header=T,sep="\t")
genodata=read.csv("re_analysis.csv",header=T,sep=",")
geno=genodata[,2:ncol(genodata)]
rownames(geno)=genodata[,1]
geno=geno[,order(colnames(geno))]
geno=as.matrix(geno)

C2N=finction(geno) {

     num=c()
     for(i in 1:nrow(geno)) {
       num[i]=length(unique(geno[i,]))
     }
     idnum=seq(1:length(num))
     dat=cbind(idnum,num)
     newdat=dat[dat[,2]==3,]
## newdat=newdat[newdat[,2]<4,]
     SNPcode=newdat[,1]
     geno_data=geno[SNPcode,]
     df1=as.data.frame(t(geno_data))
     convert <- function(x) as.integer(factor(x, levels = names(sort(-table(x)))))
     df2 <- as.data.frame(lapply(df1, FUN = convert))
     rownames(df2)=rownames(df1)
     tdf=t(df2)
     tdf=tdf-1

   return (tdf)
 }
```
